# Treatment of poorly differentiated neuroendocrine tumours with etoposide and cisplatin

**DOI:** 10.1038/sj.bjc.6690325

**Published:** 1999-12

**Authors:** E Mitry, E Baudin, M Ducreux, J-C Sabourin, P Rufié, T Aparicio, P Lasser, D Elias, P Duvillard, M Schlumberger, P Rougier

**Affiliations:** Departments of Gastroenterology, Nuclear Medicine, Pathology, Pneumology andSurgery, Institut Gustave Roussy, Villejuif, France; Department of Gastroenterology and Digestive Oncology, Hôpital Ambroise Paré, Boulogne, France

**Keywords:** neuroendocrine carcinoma, treatment, retrospective study

## Abstract

The purpose of this study was to evaluate by a retrospective analysis of 53 patients the efficacy of chemotherapy combining etoposide and cisplatin in the treatment of neuroendocrine tumours. The regimen was a combination of etoposide 100 mg m^–2^ day^–1^ for 3 days and cisplatin 100 mg m^–2^ on day 1, given by 2-h intravenous infusion, administered every 21 days. Twelve patients had a well-differentiated and 41 a poorly differentiated neuroendocrine tumour. Toxicity of treatment was assessed in 50 patients and efficacy in 52 patients. Among the 11 patients with a well-differentiated tumour evaluable for tumoural response, only one (9.4%) had a partial response for 8.5 months. Forty-one patients with a poorly differentiated tumour showed an objective response rate of 41.5% (four complete and 13 partial responses); the median duration of response was 9.2 months, the median overall survival 15 months and the median progression-free survival 8.9 months. Haematological grade 3–4 toxicity was observed in 60% of the cases with one treatment-related death, digestive grade 3–4 toxicity in 40% and grade 3 alopecia was constant. No severe renal, hearing and neurological toxicities were observed (grade 1 in 6%, 14%, 72% respectively and no grade >1). We confirm that poorly differentiated neuroendocrine tumours are chemosensitive to the etoposide plus cisplatin combination. However, the prognosis remains poor with a 2-year survival lower than 20% confirming that new therapeutic strategies have to be developed. © 1999 Cancer Research Campaign


*British Journal of Cancer* (1999) **81**(8), 1351–1355
© 1999 Cancer Research Campaign
Article no. bjoc.1999.0851

### Treatment of poorly differentiated neuroendocrine tumours with etoposide and cisplatin

**E Mitry[1,6], E Baudin[2], M Ducreux[1], J-C Sabourin[3], P Rufié[4], T Aparicio[1], P Lasser[5], D Elias[5], P Duvillard[3], M Schlumberger[2] and P Rougier[1,6]**

Departments of [1]Gastroenterology, [2]Nuclear Medicine, [3]Pathology, [4]Pneumology and [5]Surgery,. Institut Gustave Roussy, Villejuif, France; [6]Department of Gastroenterology and Digestive Oncology, Hôpital Ambroise Paré, Boulogne, France

**Summary** The purpose of this study was to evaluate by a retrospective analysis of 53 patients the efficacy of chemotherapy combining etoposide and cisplatin in the treatment of neuroendocrine tumours. The regimen was a combination of etoposide 100 mg $m^{-2}$ $day^{-1}$ for 3 days and cisplatin 100 mg $m^{-2}$ on day 1, given by 2-h intravenous infusion, administered every 21 days. Twelve patients had a well-differentiated and 41 a poorly differentiated neuroendocrine tumour. Toxicity of treatment was assessed in 50 patients and efficacy in 52 patients. Among the 11 patients with a well-differentiated tumour evaluable for tumoural response, only one (9.4%) had a partial response for 8.5 months. Forty-one patients with a poorly differentiated tumour showed an objective response rate of 41.5% (four complete and 13 partial responses); the median duration of response was 9.2 months, the median overall survival 15 months and the median progression-free survival 8.9 months. Haematological grade 3–4 toxicity was observed in 60% of the cases with one treatment-related death, digestive grade 3–4 toxicity in 40% and grade 3 alopecia was constant. No severe renal, hearing and neurological toxicities were observed (grade 1 in 6%, 14%, 72% respectively and no grade >1). We confirm that poorly differentiated neuroendocrine tumours are chemosensitive to the etoposide plus cisplatin combination. However, the prognosis remains poor with a 2-year survival lower than 20% confirming that new therapeutic strategies have to be developed. © 1999 Cancer Research Campaign

**Keywords:** neuroendocrine carcinoma; treatment; retrospective study

Despite common pathological features, gastroenteropancreatic (GEP) neuroendocrine tumours (NET) are a heterogeneous group of tumours arising from diverse sites, presenting with different clinical syndromes or biological activity, different aggressiveness and prognosis. Up to now, treatment of GEP NET has been challenging, especially when the tumours are metastatic, since data concerning the prognosis factors are still scarce. Age, tumour size, stage and primary site may be related to the outcome of NET (Johnson et al, 1983; McDermott et al, 1994; Greenberg et al, 1987; Modlin and Sandor, 1997). According to Warren and Gould's classification (Gould et al, 1983; Warren et al, 1989), well-differentiated, moderately differentiated and poorly differentiated NET should also be distinguished since this classification has a therapeutic and prognostic impact. Finally, biological activity of GEP NET may also have an impact on survival (Janson et al, 1997; Baudin et al, 1999). Chromogranin A (CgA) has been recently shown to be an independent prognosis factor of midgut NET (Janson et al, 1997). However, we have demonstrated that CgA level was independently correlated with tumour burden but also with biological activity of NET (Baudin et al, 1998). Treatment options should take into account these parameters.

*Received 5 November 1998*
*Revised 8 April 1999*
*Accepted 17 June 1999*

*Correspondence to:* E Mitry, Service d'hépatogastroentérologie et oncologie digestive, Hôpital Ambroise Paré, 9 avenue Charles de Gaulle, 92104 Boulogne Cedex, France

Surgery is the only curative modality of GEP NET, but in cases of unresectable tumour many options are available. In well-differentiated NET, careful observation may be the best attitude for patients with indolent, non-functional and slow-growing metastatic tumour. In case of progressive well-differentiated NET chemotherapy and/or biotherapies (interferon, somatostatin analogues) and/or local treatments (arterial ligation, chemo-embolization) may provide an effective palliation of symptoms and allow improvements in the quality of life in symptomatic tumours and in survival in pancreatic GEP NET (Oberg, 1994).

In contrast to well-differentiated NET, the aggressiveness of poorly differentiated NET is similar to small-cell lung cancer (SCLC), resulting in a median survival of 6 months without treatment (Johnson et al, 1983; Staren et al, 1988; Rindi et al, 1996). Most patients have metastatic disease and poor condition at the time of diagnosis and cannot be approached surgically with curative intent (Hainsworth et al, 1988; Pelley and Bukowski, 1997). In 1991, Moertel et al reported their experience with a regimen combining etoposide (VP16) and cisplatin (CDDP) (Moertel et al, 1991). A major therapeutic activity was found in 18 patients with poorly differentiated NET with an objective response (OR) rate of 67% and a median duration of response of 8 months. In contrast, the OR rate in 27 patients with well-differentiated NET was only 7%. Since this publication, the association of VP16 and CDDP has been considered as the reference treatment for poorly differentiated NET. However, confirmatory studies are still lacking. Due to the rarity of the NET, retrospective analyses are justified to further assess its anti-tumoural efficacy and to define prognostic factors.

### PATIENTS AND METHODS

### Patients

Fifty-three patients (36 males, 17 females) were treated at the Gustave-Roussy Institute with a VP16–CDDP combination between November 1988 and April 1997.

Criteria of eligibility were histologically confirmed, measurable and inoperable NET. Systematic pathological review of histologic material was performed before chemotherapy by a panel of pathologists (coordinated by JCS) and patients were classified as having a well-differentiated or poorly differentiated NET according to the Warren and Gould classification (Gould et al, 1983; Warren et al, 1989). All tumours disclosed NET morphological features including regular cells, normochromatic nucleus and eosinophilic cytoplasm arranged in ribbons, nests or sheets separated by a fine fibrovascular stroma. An immunohistochemical study with neuron-specific enolase (NSE), CgA and synaptophysin antibodies (Dako, Gioostsup, Denmark) was performed when the morphological structure precluded an unequivocal diagnosis of NET. GEP NET were classified according to their primary site as foregut (head and neck, respiratory tract, pancreas, stomach, duodenum), midgut (ileum, appendix, right colon) and hindgut (left colon, rectum, uterus) (Williams and Sandler, 1963). Patients with mixed tumours and small-cell lung carcinomas were excluded. Patients with neutropenia < 1500 $mm^{-3}$ thrombocytopenia < 100 000 $mm^{-3}$, serum creatinine >125 $mg^{-1}$ or uncontrolled infection were excluded from the study.

The staging procedures performed before starting treatment included a physical examination, biochemical profile, chest X-ray, abdominal ultrasound and thoraco-abdominal computerized tomography (CT) scan. Since 1993, In-111-DTPA-octreotide scintigraphy (octreoscan) has been systematically performed in well-differentiated NET, but only in a few patients with poorly differentiated NET considering its low sensitivity in these patients in our experience (data not shown). Additional procedures (digestive endoscopy, bronchoscopy, brain and/or bone CT scan, bone scintigraphy) were carried out according to clinical presentation and tumour location. Since 1993, hormonal tumour marker screening was standardized as described previously (Baudin et al, 1999). Briefly, NSE or CgA, 5-hydroxyindolacetic acid (5-HIAA), calcitonin (CT) and glycoprotein α-subunit (αGP) were measured in foregut-derived NET, only NSE or CgA and 5-HIAA measurements in midgut-derived NET. Hormonal hypersecretion was defined as values greater or equal to twice the upper limit of normal range found on two consecutive determinations.

### Treatment

Patients received the following chemotherapy every 21 days: CDDP, 100 $mgm^{-2}$ intravenously on day 1 in a 2-h infusion given with pre- and post-hydration, and VP16, 100 mg $m^{-2}$ $day^{-1}$ intravenously from day 1 to day 3 in a 2-h infusion. On day 1, the VP16 was started after the CDDP infusion. In case of severe neutropenia (< 1500 $mm^{-3}$) or thrombocytopenia (< 100 000 $mm^{-3}$) treatment was delayed for 1 week and doses reduced by 25%. The CDDP was not administered when creatinine clearance was > 50 ml $min^{-1}$. Therapy was continued until tumour progression or as long as the therapy was well-tolerated. Appropriate anti-emetics (anti-HT3) were administered with each course of therapy. Concurrent sandostatin was allowed during chemotherapy, if necessary.

### Response assessment

Tumoral objective response (OR) was evaluated every three courses during the treatment period and every 1–3 months thereafter with physical examination of patient and appropriate imaging (ultrasound and/or CT scan) and laboratory studies. According to WHO criteria, a complete OR was defined as a total disappearance of all detectable tumours. A partial OR was defined as a greater than 50% reduction in the product of the longest perpendicular diameters of measurable lesions for at least 4 weeks in the absence of new lesions or the progression of existing lesions. Stable disease was defined as a reduction of tumour size of less than 50%, or an increase in tumour size of less than 25%. Progression was defined as a greater than 25% increase in measurable disease or the development of new metastases. To declare a hormonal response, it was required that this parameter be reduced to less than 50% of the pretreatment value or to normal range. Time to progression was the time from day 1 of the treatment to the time when a progression was detected. Duration of objective responses was measured from day 1 of the treatment to the time of progression or censoring. Patient survival was the time from day 1 of the treatment to time of death or censoring.

To evaluate prognostic factors that influenced response to treatment or survival, the following parameters were analysed in poorly differentiated NET: age, gender, primary tumour site, hormonal secretions (defined as present or absent), prior therapies and disease extension defined as limited or extensive stage. An extensive stage was defined as a disease that had spread beyond loco regional boundaries.

Toxicity was assessed after each course of chemotherapy by physical examination, direct questioning, measurement of haematological and biochemical parameters and graded according to the WHO criteria (Miller et al, 1981).

### Statistical analysis

Statistical analyses were performed using the BMDP statistical software. Comparison of qualitative variables were made by the Fisher's exact test and comparison of quantitative variables by *t*-tests. The survival function for time to progression and time to death was estimated using the Kaplan–Meier method (Kaplan and Meier, 1958) and the log-rank statistic was used to compare survival distributions (Mantel and Haenszel, 1959). Differences were considered significant at a P-value of less than 0.05.

### RESULTS

The main characteristics of the patients are summarized in Table 1. Twelve patients (four males, eight females) had a well-differentiated NET and 41 (32 males, nine females) a poorly differentiated NET.

Eight patients (one well-differentiated, seven poorly differentiated) had tumours with unknown primary site. When the primary tumour site was known, it was largely dependent upon differentiation: 94% of the poorly differentiated tumours compared to 54.5% of the well-differentiated tumours originated from the foregut. Only nine tumours, one (8.3%) well-differentiated and eight (19.5%) poorly differentiated (pancreas: three; respiratory tract: two; mediastinum: two; head and neck: one), had a limited stage, whereas the other 44 were metastatic. Abnormal hormonal secretion was found in 64% of well-differentiated and in 47% of poorly differentiated tumours.

 *© 1999 Cancer Research Campaign*

**Table 1** Patient characteristics

| | Well-differentiated tumours | Poorly differentiated tumours | *P*-value |
|---|---|---|---|
| Total number of patients | 12 | 41 | |
| Male/female | 4/8 | 32/9 | 0.01 |
| Median age in years (range) | 46.5 (26–62) | 53.4 (20–76) | |
| Stage | | | |
| Limited stage | 1 | 8 | NS |
| Extensive stage | 11 | 33 | |
| Primary tumour site | | | |
| Foregut | 6 | 32 | 0.009 |
| Pancreas | 4 | 13 | |
| Stomach | 0 | 3 | |
| Gallbladder | 0 | 2 | |
| Respiratory tract | 2 | 5 | |
| Mediastinum | 0 | 5 | |
| Head and neck | 0 | 4 | |
| Midgut | 4 | 2 | |
| Small bowel | 3 | 0 | |
| Appendix | 1 | 0 | |
| Right colon | 0 | 2 | |
| Hindgut | 1 | 0 | |
| Uterus | 1 | 0 | |
| Unknown Primary site | 1 | 7 | |
| Abnormal hormonal secretion[a] | 7 (64%) | 16 (47%) | NS |
| Prior treatment | 9 (75%) | 24 (58%) | NS |
| Surgery | 8 | 13 | |
| Chemotherapy | 7 | 13 | |
| Sandostatin | 2 | 3 | |
| Median time between diagnosis and start of the treatment (range)[b] | 8.0 (1.4–28.7) | 3.0 (0–43.9) | |
| Median number of courses (range) | 4 (1–8) | 6 (1–9) | |

[a]Data available for 45 patients, [b]in months.

**Table 2** Treatment results and survival

| | Well-differentiated tumours *n* (%) | Poorly differentiated tumours *n* (%) | *P*-value |
|---|---|---|---|
| Tumoural response | | | |
| Complete regression | 0 | 4 (9.8%) | 0.09 |
| Partial regression | 1 (9.1%) | 13 (31.7%) | |
| Stable | 4 (36.4%) | 14 (34.1%) | |
| Progression | 6 (54.5%) | 10 (24.4%) | |
| Median duration of response (range)[9] | 8.5 | 9.24 (4.5–23.5) | 0.36 |
| Survival | | | |
| Median survival (range)[a,b] | 17.6 (8.6–72+) | 15 (11.7–25) | 0.18 |
| Median time to progression (range)[a,b] | 2.3 (0.9–12.1) | 8.9 (6.7–13.4) | 0.3 |

[a]In months, [b]Kaplan–Meier method.

The VP16 + CDDP combination was given as first-line chemotherapy in 41.7% (5/12) of well-differentiated tumours and in 70.7% (29/41) of poorly differentiated tumours. Among patients with a well-differentiated tumour who received the VP16–CDDP as first line chemotherapy, one was treated before 1991; the others had an aggressive tumour initially classified as poorly differentiated but finally classified, after pathological reviewing, as well-differentiated. The median number of chemotherapy cycles was four in patients with a well-differentiated tumour (range 1–8) and six in patients with a poorly differentiated tumour (range 1–9). Two patients with poorly differentiated tumours received sandostatin (100 μg × 2 day$^{-1}$) concurrently with chemotherapy.

### Therapeutic results

One patient with a well-differentiated tumour died of a pulmonary embolism after the first course of treatment and could not be evaluated. None of the 11 evaluable patients with a well-differentiated tumour had a complete response; one (9.1%) showed a partial response, four (36.4%) stable disease and six (54.5%) a progressive disease. Among patients with a poorly differentiated tumour, four (9.8%) had a complete response, 13 (31.7%) a partial response, 14 (34.1%) stable disease and ten (24.4%) progressive disease. The overall OR rate was 41.5% (17/41) among patients with a poorly differentiated tumour and 9.1% (1/11) among patients with a well-differentiated tumour. This difference was not significant ($P = 0.09$) (Table 2).

Response to treatment occurred quickly. All the responders showed an OR at the first therapeutic evaluation. The median duration of tumoural response was 8.5 months for the patient with a well-differentiated tumour and 9.2 months (range 4.5–23.5) for patients with poorly differentiated tumours. The duration of tumoural response was respectively 10.6, 10.7, 12.3 and 13.3 months for the patients with complete response.

No relationship was found between response and patient age or gender, primary tumour site, stage, hormonal secretions, prior treatment or chemotherapy line (Table 3). It should be mentioned that the response rate among poorly differentiated tumours of unknown primary site was lower compared to tumours with a known primary site (1/7 14.3% vs 16/34 47.1%, $P = 0.2$). Among patients with poorly differentiated tumours who had abnormal hormonal secretion, 87.5% of tumoural responses were accompanied by a hormonal response. It is noteworthy that 25% of the hormonal responders showed no tumoural response.

### Survival

After a median follow-up of 64 months (range 20–111) for patients with a well-differentiated tumour and 36 months (range 6–68) for patients with a poorly differentiated tumour ($P = 0.001$), eight (66%) patients with a well-differentiated tumour and 26 (63%) with a poorly differentiated tumour had died, two (16.6%) and four (9.7%) were alive with progressive disease, one (8.3%) and five (12.2%) were alive with stable disease, one (8.3%) and two (4.8%) were alive in complete remission. Four patients with poorly differentiated pancreatic tumours were lost to follow-up at 5, 34, 41 and 52 weeks after the beginning of treatment. None of them was a responder and all had progressive disease when they were seen for the last time.

Median survival for well-differentiated tumours was 17.6 months (range 8.6–72, mean = 32.5 months) and 15 months (range 11.7–25) for poorly differentiated tumours. Median progression-free survival was 2.3 months (range 0.9–12.1) for

© 1999 Cancer Research Campaign

**Table 3** Characteristics of patients with poorly differentiated tumours according to tumoral response

| Variables | Responders | Non-responders | *P*-value |
|---|---|---|---|
| Sex | | | |
| Male | 15 (88.2%) | 17 (70.8%) | 0.26[a] |
| Female | 2 (11.8%) | 7 (29.2%) | |
| Age (years) | | | |
| ≤60 | 13 (76.5%) | 13 (54.2%) | 0.19[a] |
| >60 | 4 (23.5%) | 11 (45.8%) | |
| Primary tumour site | | | |
| Foregut | 15 (46.9%) | 17 (53.1%) | 0.2[a] |
| Midgut | 1 (50%) | 1 (50%) | |
| Unknown | 1 (14.3%) | 6 (85.7%) | |
| Stage | | | |
| Limited | 3 (17.6%) | 5 (20.8%) | 1.00[a] |
| Extended | 14 (82.4%) | 19 (79.2%) | |
| Abnormal hormonal secretion | | | |
| Yes | 7 (46.7%) | 11 (57.9%) | 0.73[a] |
| No | 8 (53.3%) | 8 (42.1%) | |
| Prior chemotherapy | | | |
| No | 12 (70.6%) | 17 (70.8%) | 1.00[a] |
| Yes | 5 (29.4%) | 7 (29.2%) | |
| Median survival (range)[b] | 16.2 (9.6–) | 13.1 (8.4–25.0) | 0.3[c] |
| Median progression-free survival (range)[b] | 10.6 (8.3–16.2) | 7.4 (2.5–16.3) | 0.4[c] |

[a]Fisher's exact test, [b]in months, [c]log-rank test.

**Table 4** Treatment toxicities

| Toxicity | Total (%) *n* = 50 | Grade 1–2 | Grade 3–4 |
|---|---|---|---|
| Non-haematological toxicity | | | |
| Nausea–vomiting | 38 (76%) | 18 | 20 |
| Neuropathy | 36 (72%) | 36 | 0 |
| Hearing loss | 7 (14%) | 7 | 0 |
| Renal toxicity | 3 (6%) | 3 | 0 |
| Haematological toxicity | | | |
| Leukopenia | 36 (72%) | 15 | 21 |
| Neutropenia | 35 (70%) | 5 | 30[a] |
| Thrombocytopenia | 12 (24%) | 6 | 6 |
| Anaemia | 16 (32.7%) | 10 | 6 |

[a]Including eight cases of febrile aplasia and one toxic death.

well-differentiated and 8.9 months (range 6.7–13.4) for poorly differentiated tumours (Table 2).

Among poorly differentiated tumours, there was a trend for a better overall survival and better progression-free survival among responders, but statistical significance was not reached (Table 3). No variable was significantly associated with survival.

### Toxicity

A total of 256 courses of treatment was completed. Fifty patients were evaluated for drug toxicity (one patient died of a pulmonary embolism after the first course of treatment and two patients received some cycles in other centres and data about drug toxicity were incomplete). Severe toxicity required cessation of treatment in only one patient (1.9%). Grade 3 or 4 nausea and vomiting occurred in 40% of the cases. Sixty per cent (30/50) of the patients had severe neutropenia and 16% febrile aplasia. One patient died of septic shock during aplasia. Severe anaemia and thrombocytopenia occurred in 12% of the cases. With the exception of alopecia, there were no other severe toxicities. Neurological toxicity grade 1 was frequent after four courses of treatment. Hearing and renal toxicities grade 1 were rare (Table 4).

### DISCUSSION

The combination of VP16 and CDDP is an ineffective treatment for well-differentiated NET: no complete response and only 9.1% of partial responses were observed. These results are in agreement with Moertel et al who reported 7% of partial responses among 27 patients (Moertel et al, 1991). In this group, the median time from diagnosis to start of treatment was only 8 months, the median overall survival 17.6 months and the mean overall survival 32 months. This survival is very low for well-differentiated tumours. This could probably be explained by the fact that well-differentiated NET of this study which received the VP16–CDDP combination were selected because of their aggressiveness. Thus, the prognosis of this selected group is poorer and not representative of the prognosis of well-differentiated NET in general.

The high chemosensibility of poorly differentiated NET is confirmed: the OR rate was 41.5% with 9.8% of complete responses. These results seem to be less favourable than those reported previously in smaller series: Moertel et al observed 67% of OR with 17% of complete responses among 18 patients (Moertel et al, 1991) and Seitz et al observed 75% of OR with 25% of major responses among eight patients (Seitz et al, 1995). The size of the present study population was larger than that of the other series, therefore our estimates of tumoural response and survival are probably more precise. The calculated 95% confidence interval of the OR rate was between 45% and 89% for Moertel et al, and from 26% to 57% in the present study.

Moertel et al used a 24-h intravenous infusion regimen in order to enhance the therapeutic interaction of VP16 and CDDP (Moertel et al, 1991). One could argue that a rapid injection regimen would be less effective, but results comparable to our infusion regimen were reported with rapid injection regimen (Hainsworth et al, 1988).

Response to treatment occurred early and it is probably unnecessary to continue chemotherapy for patients who have not responded after three courses. Nevertheless, considering the aggressiveness of this type of tumour and the absence of alternative efficient therapy, a stabilization for patients with progressive disease could be considered as a positive result. In this situation, continuing the chemotherapy, if it is well tolerated, may be beneficial.

We observed a particularly low response rate (14.3%) for poorly-differentiated neuroendocrine carcinomas of unknown primary site. In contrast, Hainsworth et al (1988) have reported 72% of major responses among 23 patients treated by VP16–CDDP combination or other CDDP-based regimens.

Response to treatment increased the overall survival and the progression-free survival by 3 months. With chemotherapy, median overall survival was of 15–19 months compared to 6–7 months without treatment in the literature (Johnson et al, 1983; Staren et al, 1988; Rindi et al, 1996).

Haematological and neurological toxicity were a major problem with this chemotherapy regimen. Seitz et al failed to avoid the

 © 1999 Cancer Research Campaign complications by the systematic use of granulocyte colony-stimulating factor (Seitz et al, 1995). The regimen we used was less intensive and better tolerated with less acute toxic effects. The treatment was stopped in only one patient because of severe toxicity and we observed one toxic death. Cumulative toxicity was more frequent, but less severe compared to Moertel's results. This was probably related to the higher median number of courses.

We conclude that GEP NET differentiation is a main prognosis factor which should be clearly specified when determining a therapeutic strategy. Poorly differentiated NET are characterized by rapid tumour growth and chemosensitivity. Chemotherapy with VP16 plus CDDP probably improves survival of patients with such tumours but the prognosis remains poor: most of the patients relapsed quickly and the 2-year survival is lower than 20%. Other therapeutic approaches should be developed.

### ACKNOWLEDGEMENTS

We would like to thank Solange Delannay for her help and Ingrid Kuchental for proofreading the manuscript.

### REFERENCES

Baudin E, Gigliotti A, Ducreux M, Ropers J, Comoy E, Sabourin JC, Bidart JM, Cailleux AF, Bonacci R, Ruffie P and Schlumberger M (1998) Neuron-specific enolase and chromogranin A as markers of neuroendocrine tumours. *Br J Cancer* **78**: 1102–1107

Baudin E, Bidart JM, Rougier P, Lazar V, Ruffie P, Ropers J, Ducreux M, Troalen F, Sabourin JC, Comoy E, Lasser P, DeBaere T and Schlumberger M (1999) Screening for multiple endocrine neoplasia type 1 and hormonal production in apparently sporadic neuroendocrine tumors. *J Clin Endocrinol Metab* **84**: 69–75

Gould VE, Linnoila RI, Memoli VA and Warren WH (1983) Neuroendocrine cells and neuroendocrine neoplasms of the lung. *Pathol Annu* **18**: 287–330

Greenberg RS, Baumgarten DA, Clark WS, Isacson P and McKeen K (1987) Prognostic factors for gastrointestinal and bronchopulmonary carcinoid tumors. *Cancer* **60**: 2476–2483

Hainsworth JD, Johnson MH and Greco A (1988) Poorly differentiated neuroendocrine carcinoma of unknown primary site. *Ann Intern Med* **109**: 364–371

Janson ET, Holmberg L, Stridsberg M, Eriksson B, Theodorsson E, Wilander E and Oberg K (1997) Carcinoid tumors: analysis of prognostic factors and survival in 301 patients from a referral center. *Ann Oncol* **8**: 685–690

Johnson LA, Lavin P, Moertel CG, Weiland L, Dayal Y, Doos WG, Geller SA, Cooper HS, Nime F, Masse S, Simson IW, Sumner H, Folsch E and Engstrom P (1983) Carcinoids: the association of histologic growth pattern and survival. *Cancer* **51**: 882–889

Kaplan EL and Meier P (1958) Non-parametric estimation for incomplete observations. *J Am Stat Assoc* **53**: 457–481

McDermott EW, Guduric B and Brennan MF (1994) Prognostic variables in patients with gastrointestinal carcinoid tumours. *Br J Surg* **81**: 1007–1009

Mantel N and Haenszel W (1959) Statistical aspects of the analysis of data from retrospective studies of disease. *J Natl Cancer Inst* **22**: 719–748

Miller AB, Hoogstraten B, Staquet M and Winkler A (1981) Reporting results of cancer treatment. *Cancer* **47**: 207–214

Modlin IM and Sandor A (1997) An analysis of 8305 cases of carcinoid tumors. *Cancer* **79**: 813–829

Moertel CG, Kvols LK, O'Connell MJ and Rubin J (1991) Treatment of neuroendocrine carcinomas with combined etoposide and cisplatin. *Cancer* **68**: 227–232

Oberg K (1994) Treatment of neuroendocrine tumors. *Cancer Treat Rev* **20**: 331–355

Pelley RJ and Bukowski RM (1997) Recent advances in diagnosis and therapy of neuroendocrine tumors of the gastrointestinal tract. *Curr Opin Oncol* **9**: 68–74

Rindi G, Bordi C, Rappel S, Larosa S, Stolte M and Solcia E (1996) Gastric carcinoids and neuroendocrine carcinomas: pathogenesis, pathology, and behavior. *World J Surg* **20**: 168–172

Seitz J, Perrier H, Giovannini M, Monges G, Fourdan O, Barrière N and Viens P (1995) Cancers neuroendocrines anaplasiques avancés: intérêt de l'association VP16-CDDP. *Bull Cancer* **82**: 433

Staren ED, Gould VE, Warren WH, Wool NL, Bines S, Baker J, Bonomi P, Roseman DL and Economou SG (1988) Neuroendocrine carcinomas of the colon and rectum: a clinicopathologic evaluation. *Surgery* **104**: 1080–1089

Warren WH, Faber LP and Gould VE (1989) Neuroendocrine neoplams of the lung: a clinicopathologic update. *J Thorac Cardiovasc Surg* **98**: 321–332

Williams ED and Sandler M (1963) The classification of carcinoid tumors. *Lancet* **1**: 238

*© 1999 Cancer Research Campaign*
